# Chronic Psychological Stress Disrupted the Composition of the Murine Colonic Microbiota and Accelerated a Murine Model of Inflammatory Bowel Disease

**DOI:** 10.1371/journal.pone.0150559

**Published:** 2016-03-07

**Authors:** Yohei Watanabe, Sohei Arase, Noriko Nagaoka, Mitsuhisa Kawai, Satoshi Matsumoto

**Affiliations:** Yakult Central Institute, Kunitachi, Tokyo, Japan; INSERM, FRANCE

## Abstract

The effect of psychological stress on the gastrointestinal microbiota is widely recognized. Chronic psychological stress may be associated with increased disease activity in inflammatory bowel disease, but the relationships among psychological stress, the gastrointestinal microbiota, and the severity of colitis is not yet fully understood. Here, we examined the impact of 12-week repeated water-avoidance stress on the microbiota of two inbred strains of T cell receptor alpha chain gene knockout mouse (background, BALB/c and C57BL/6) by means of next-generation sequencing of bacterial 16S rRNA genes. In both mouse strains, knockout of the T cell receptor alpha chain gene caused a loss of gastrointestinal microbial diversity and stability. Chronic exposure to repeated water-avoidance stress markedly altered the composition of the colonic microbiota of C57BL/6 mice, but not of BALB/c mice. In C57BL/6 mice, the relative abundance of genus *Clostridium*, some members of which produce the toxin phospholipase C, was increased, which was weakly positively associated with colitis severity, suggesting that expansion of specific populations of indigenous pathogens may be involved in the exacerbation of colitis. However, we also found that colitis was not exacerbated in mice with a relatively diverse microbiota even if their colonic microbiota contained an expanded phospholipase C-producing *Clostridium* population. Exposure to chronic stress also altered the concentration of free immunoglobulin A in colonic contents, which may be related to both the loss of bacterial diversity in the colonic microbiota and the severity of the colitis exacerbation. Together, these results suggest that long-term exposure to psychological stress induces dysbiosis in the immunodeficient mouse in a strain-specific manner and also that alteration of microbial diversity, which may be related to an altered pattern of immunoglobulin secretion in the gastrointestinal tract, might play a crucial role in the development of chronic stress-induced colitis.

## Introduction

Recent advances in DNA sequencing technology have revealed that several hundred species of bacteria colonize the human gastrointestinal (GI) tract [1]. The composition and biological function of the GI microbiota influence the host immune system and are therefore linked to host health and disease, and the GI microbiota is now recognized as an “organ” within the human body [2]. The roles of the GI microbiota in various diseases has been widely recognized, such as colorectal cancer [3], type 1 diabetes [4], obesity [5], and inflammatory bowel disease (IBD) [6].

IBD (Crohn’s disease and ulcerative colitis) is a complex chronic inflammatory disorder of the GI tract that is a major public health problem in many countries [7]. Long-standing IBD confers an increased risk of colorectal cancer [8]. A defining characteristic of the chronic inflammation associated with IBD is that it follows a recurrent course of repeated periods of active disease followed by remission. The etiology of IBD is unknown, but chronic psychological stress may increase disease activity in IBD [9].

Psychological stress is known to affect the GI microbiota [10]. For example, exposure to a social stressor for two hours has been shown to alter the community structure of the colonic mucosa-associated microbiota in the cecum of mature mice [11]. Seven-day repeated exposure to restraint stress has also been shown to reduce microbial species richness and diversity and increase susceptibility to colonization by pathogenic bacteria in the murine intestine [12]. Six-day repeated exposure to a social stressor has been shown to induce increases or decreases in the relative abundances of certain bacterial genera, with some of these changes being significantly correlated with alterations in the circulating levels of proinflammatory cytokines in the intestinal microbiota of mice [13]. Finally, ten-day repeated exposure of mice to water-avoidance stress (rWAS) has been shown to induce the production of corticotropin-releasing hormone, leading to inhibition of the nucleotide-binding oligomerization domain protein-like receptor family pyrin domain containing 6 inflammasome, which alters the composition of the microbiota [14].

Together, these studies indicate that psychological stress influences both the function and microbiota of the GI tract via the hypothalamic–pituitary–adrenal axis and nervous system [15]. However, the exposure period to psychological stress in these previous studies was less than 10 days; therefore, the impact of chronic psychological stress on the GI microbiota is not yet fully understood. People in modern society are often exposed to psychological stress for periods of months or even years, meaning that studies using realistically long periods of psychological stress are necessary to further our understanding of the effects of long-term stress on the GI microbiota and the development of colonic inflammation.

Here, we investigated the impact of 12-week rWAS on the GI microbiota and development of colonic inflammation in T cell receptor alpha chain gene (*Tcra*) knockout mice with the genetic backgrounds C57BL/6 and BALB/c, which spontaneously develop human ulcerative colitis-like chronic colitis [16] and show a differential response to stress in the gut [17]. A strain-specific response to rWAS was observed, and a significant change in the microbial composition of the colonic microbiota was observed in mice that developed severe colitis. Cell number of indigenous *Clostridium perfringens*, a phospholipase C–producing pathogen, as assessed by quantitative polymerase chain reaction (PCR), was weakly positively correlated with the severity of colitis, as assessed by histological scoring, whereas species richness of the colonic microbiota, as assessed by phylogenetic diversity, was strongly negatively correlated with the severity of colitis. Changes in the concentrations of free immunoglobulin A (IgA) in colonic contents was also observed.

## Materials and Methods

### Animals

*Tcra*^−/−^ homozygous mice with the C57BL/6 (B6) (B6.129P2-*Tcra*^*tm1Mom*^/Yit; B6-*Tcra*^−/−^) or BALB/c (B/c) (C.129P2(B6)-*Tcra*^*tmlMom*^/Yit; B/c-*Tcra*^−/−^) background were kindly provided by Dr. Susumu Tonegawa (Massachusetts Institute of Technology, Cambridge, MA). These mice were then bred with C57BL/6 or BALB/c mice from our own colony to obtain heterozygous mice. All mice were maintained in the animal facility at Yakult Central Institute, and all procedures involving animals described in this study were approved by the Animal Experimental Committee of Yakult Central Institute (approval numbers 13–0091 and 13–0173).

### rWAS

Mice (age, 8 weeks) were put on a small platform surrounded by water for one hour per day for one day (low-frequency rWAS group; LFW) or five days (high-frequency rWAS group; HFW) per week. After 12 weeks of exposure to rWAS, the mice (age, 20 weeks) were euthanized and the colonic content of each mouse was collected and stored at −80°C until use. The histological score for colitis was estimated in a blinded fashion by summing the scores of six types of histological change in each mouse colon (Table 1). *Tcra*^−/−^ mice not exposed to rWAS were used as controls (CON). *Tcra*^−/+^ heterozygous mice not exposed to rWAS (HET) were also used to investigate the impact of T-cell receptor α-chain deficiency on the colonic microbiota. The mice of HET, CON, LFW and HFW groups were housed in different cages respectively in a same laboratory. To help clarify the relationship between the colonic microbiota and exacerbation of colitis, we combined the B6-LFW and B6-HFW mice into one group, ordered the mice by histological score, and used the median histological score as the cut-off value to divide the mice into two groups (S1 Table). The relative expression level of cytokines and stress-related mRNA in each groups were measured (S2 Table). In the same manner as B6 mice, B/c-LFW mice and B/c-HFW mice was split by histological score, but the significant difference of the overall bacterial composition and diversity, or blooming of the specific bacterial species were not confirmed in higher-inflammation group compared to lower-inflammation group (data not shown).

**Table 1:** Histological score criteria.

	Score			
Histological changes	0	1	2	3
**Active inflammation**	None	PMN accumulation (mild)	PMN accumulation (severe) without crypt abscess	PMN accumulation (severe) with crypt abscess
**Chronic inflammation**	None	MNC accumulation (mild)	MNC accumulation (severe) in LP	MNC accumulation (severe) in LP and SM
**Epithelial hyperplasia**	None	Mild	Intermediate	Moderate
**Ulcer**	None	Erosion (mild)	Erosion (severe)	Ulcer
**Malignancy**	None	LGD	HGD	Invasive cancer
**Extension**	None	Focal (30%)	60%	Entire region

PMN, polymorphonuclear leukocyte; MNC, mononuclear cell; LP, lamina propria; SM, submucosal; LGD, low-grade dysplasia; HGD, high-grade dysplasia.

### 16S rRNA gene sequencing

DNA extraction from the colonic samples was performed by using glass beads and phenol, as described previously [18]. Amplification and sequencing of the V4 region of the bacterial 16S rRNA gene was performed by using the primers 515F and 806R, as described previously [19], with the following minor modifications. Barcoded amplicons were generated in triplicate by using TaKaRa Ex Taq HS (Takara Bio, Shiga, Japan) with a final volume of 25 μL containing 10 ng of template DNA. The PCR amplification program comprised an initial denaturation at 94°C for 3 min, then 20 cycles of 94°C for 45 s, 50°C for 60 s, and 72°C for 90 s, and a final elongation step at 72°C for 5 min. The triplicated amplicons were mixed, purified with a High Pure PCR Product Purification kit (Roche Diagnostics GmbH, Mannheim, Germany), quantified with a Quant-iT PicoGreen dsDNA Assay Kit (Invitrogen, Eugene, OR, USA), pooled in equimolar amounts, and then sequenced on an Illumina MiSeq platform with a MiSeq Reagent Kit v2 (Illumina, San Diego, CA, USA), as described previously [19].

### Bioinformatics analysis

16S rRNA gene sequences were analyzed by using the Quantitative Insights Into Microbial Ecology (QIIME) software package version 1.8.0 [20]. Raw 250-bp paired-end sequence reads were combined by using the fastq-join script [21] with the minimum allowed overlap in base-pairs required to join pairs set at 150 bp, and the maximum allowed percentage difference within a region of overlap set at 15%. Quality filtering was performed as described previously, except that parameter *c* = 0.0005% [22]. Further data processing comprised filtering by clustering similar sequences with less than 3% dissimilarity by using the USEARCH algorithm version 5.2.32 [23] with an open-reference OTU (operational taxonomic unit) clustering method using the Greengenes database (13_8; greengenes.lbl.gov/), and detecting and removing chimeras by using the UCHIME algorithm [24]. The most abundant sequence in each OTU was selected as the representative sequence and the resulting OTUs were assigned to taxa by using the Ribosomal Database Project classifier [25] trained on the Greengenes reference database [26] via QIIME set at a minimum confidence score of 80%. The OTUs were aligned against the Greengenes core reference alignment by using the Python Nearest Alignment Space Termination alignment algorithm in QIIME [27]. The most closely related known species to each OTU was determined by means of a local BLAST search of the All-Species Living Tree Project database [28]. To test for statistically significant variations in the frequency of genera and OTUs between groups, we used the nonparametric *t*-test with the Benjamini–Hochberg False Discovery Rate (FDR) correction implemented in the group_significance.py script in QIIME. A phylogenetic tree was constructed by using the FastTree approximately-maximum-likelihood program in QIIME [29]. Prior to the calculation of diversity metrics, the sequence libraries were randomly subsampled to achieve an even sampling depth (75 000 reads per sample). OTU diversity within and between samples was analyzed by using the α- and β-diversity indices, respectively. Alpha diversity was measured by using the observed species metrics and whole-tree phylogenetic diversity. Alpha diversity index values were compared using a nonparametric two-sample *t*-test in the compare_alpha_diversity.py script in QIIME set at the default number of Monte-Carlo permutations (999). Beta diversity was measured by using the unweighted UniFrac pipeline in QIIME [30]. Statistically significant differences in the UniFrac distance were then tested for by using the make_distance_boxplots.py script with nonparametric options in QIIME. Principal-coordinates analysis was used to interpret and visualize the variations in the unweighted UniFrac distance matrix. The largest amount of variation is explained by the first principal coordinate and the second largest by the second principal coordinate. Sequence data were deposited in the DNA Data Bank of Japan (http://www.ddbj.nig.ac.jp/) Sequence Read Archive under BioProject Accession No. PRJDB4266.

### Quantification of *Clostridium perfringens*

*Clostridium perfringens* in colonic contents was quantified by means of quantitative PCR by using the *C*. *perfringens* phospholipase C–specific primer set described previously [31].

### Isolation of phospholipase C–producing *Clostridium* species

To isolate phospholipase C (PLC)–producing *Clostridium* species, mouse colonic contents were diluted with 0.1 M phosphate-buffered saline (pH 7) and 100 μL was spread on CW agar plates containing kanamycin and egg yolk (Nikken Biomedical Laboratory, Kyoto, Japan). Plates were incubated at 37°C for 2 days under anaerobic conditions created by using an AnaeroPack (Mitsubishi Gas Chemical, Tokyo, Japan). PLC-positive colonies surrounded by a large opalescent zone were picked and used to obtain pure cultures. DNA extraction, PCR, and 16S rRNA gene sequencing were performed as described previously [32]. Sequences with high similarity were retrieved from the National Center for Biotechnology Information database (http://www.ncbi.nlm.nih.gov/) by using the BLASTN program.

### Quantification of IgA and IgG in colonic contents

Colonic contents were weighed and 10-fold diluted with 10 mM phosphate-buffered saline (pH 7) and centrifuged. The concentrations of IgA and IgG in the serially diluted supernatant were determined by using a sandwich enzyme-linked immunosorbent assay, as previously described [33]. The Wilcoxon rank-sum test was used to test for statistical significance of the difference between groups.

## Results

### *Tcra* knockout altered the colonic microbiota composition of B/c mice

**Fig 1:**
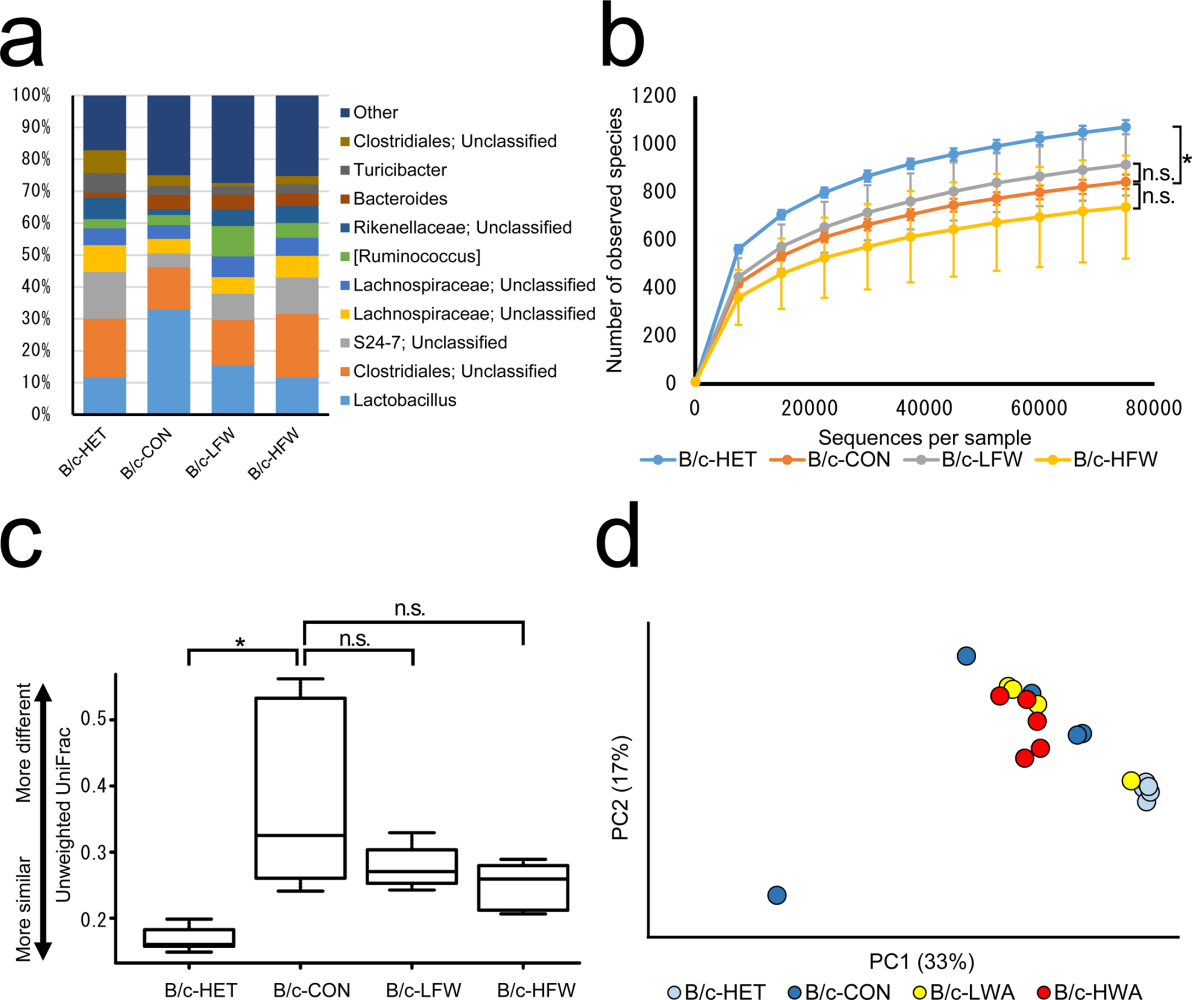
Composition of the colonic microbiota of BALB/c *Tcra*^−/−^ mice after exposure to 12-week repeated water-avoidance stress. (a) Bacterial taxonomic profiling of the colonic microbiota at the genus level. (b) Bacterial richness as represented by observed species rarefaction measured based on 1 to 75 000 sequences. Mean ± standard deviation. (c) Average unweighted UniFrac distance. (d) Principal-coordinates analysis plot based on the unweighted UniFrac distance. HET, *Tcra*^−/+^ mice not exposed to repeated water-avoidance stress (rWAS); CON, *Tcra*^*−/−*^ mice not exposed to rWAS; LFW, *Tcra*^−/−^ mice exposed to low-frequency (1 day) rWAS; HFW, *Tcra*^−/−^ mice exposed to high-frequency (5 days) rWAS. PC1 and PC2 are the first two principal coordinates. * *P* < 0.05; n.s., not significant (*P* > 0.05).

We compared the community structures of the colonic microbiota of *Tcra*^−/+^ mice (B/c-HET; *n* = 5) and of *Tcra*^−/−^ mice (B/c-CON; *n* = 5) not exposed to rWAS and found that the relative abundances of several bacterial genera were lower in the colonic microbiota of B/c-CON mice (e.g., unclassified genera of families S24-7 and *Rikenellaceae*) compared with that in B/c-HET mice (Fig 1A), but these decreases were not statistically significant (FDR-corrected *P* value = 0.09). Species richness was significantly lower (*P* < 0.05) in the colonic microbiota of B/c-CON mice compared with that of B/c-HET mice (Fig 1B). The average unweighted UniFrac distance for the colonic microbiota of B/c-CON mice was significantly larger (*P* < 0.05) than that of B/c-HET mice (Fig 1C). Together with the result of a principal-coordinates analysis plot in which the B/c-HET mice were clustered together whereas B/c-CON mice were more dispersed (Fig 1D), these results indicated that the community structure of the colonic microbiota of B/c-HET mice was more stable than that of B/c-CON mice.

### Exposure to rWAS did not induce significant changes in the colonic microbiota of B/c-*Tcra*^−/−^ mice

We investigated the impact of rWAS on the community structures of the colonic microbiota of B/c mice exposed to low- or high-frequency rWAS (LFW, *n* = 4; HFW, *n* = 5). There were no statistically significant changes in the relative abundance of any genus or in species richness (Fig 1A and 1B), and B/c-CON mice and B/c-LFW or B/c-HFW mice were almost indistinguishable in the PCoA plot (Fig 1D). Therefore, the microbial compositions of the microbiota were comparable between B/c-CON, B/c-LFW, and B/c-HFW mice.

### *Tcra* knockout altered the composition of the colonic microbiota of B6 mice

**Fig 2:**
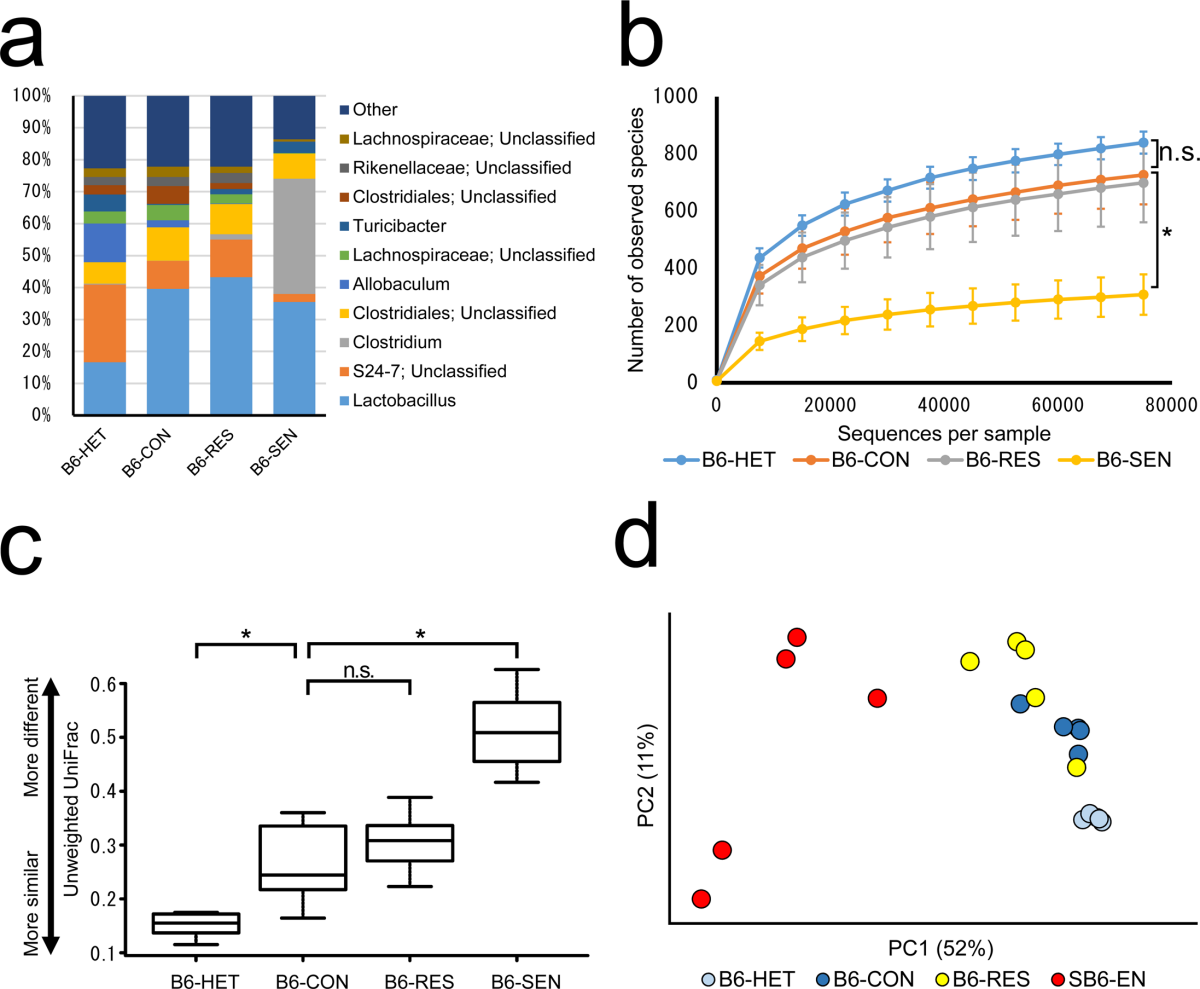
Composition of the colonic microbiota of B6 *Tcra*^−/−^ mice after exposure to 12-week repeated water-avoidance stress. (a) Bacterial taxonomic profiling of the colonic microbiota at the genus level. (b) Bacterial richness as represented by observed species rarefaction measured based on 1 to 75 000 sequences. (c) Average unweighted UniFrac distance. (d) Principal-coordinates analysis plot based on the weighted UniFrac distance. HET, *Tcra*^−/+^ mice not exposed to repeated water-avoidance stress (rWAS); CON, *Tcra*^−/−^ mice not exposed to rWAS; RES, *Tcra*^−/−^ mice resistant to rWAS-induced colitis; SEN, *Tcra*^−/−^ mice sensitive to rWAS-induced colitis. PC1 and PC2 are the first two principal coordinates. * *P* < 0.05; n.s., not significant (*P* > 0.05).

**Fig 3:**
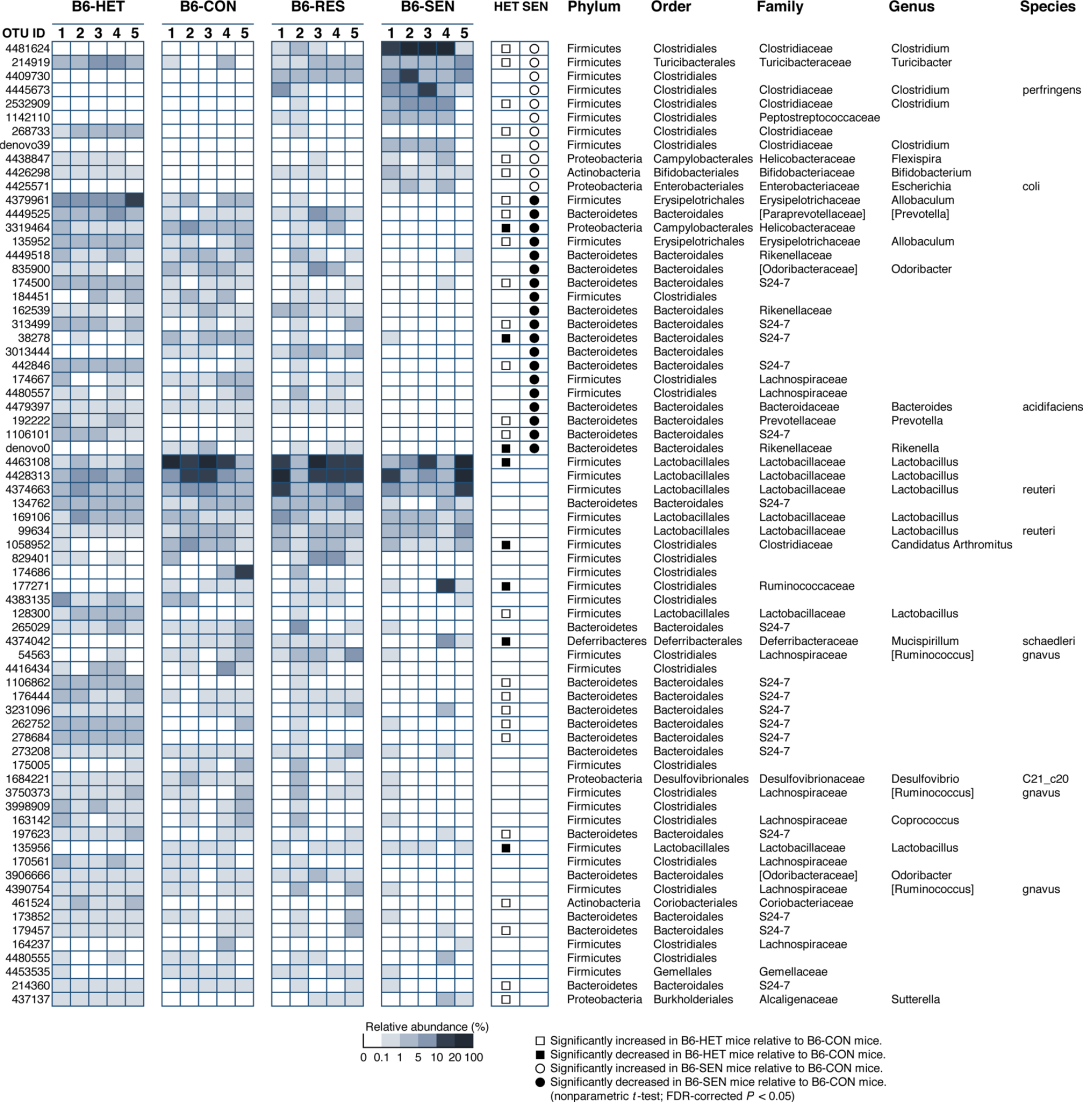
Relative abundances of operational taxonomic units (OTUs). Heatmap showing the relative abundances of 70 OTUs and their taxonomic assignment acquired by using the Ribosomal Database Project classifier. Taxa names in brackets are annotations supplied by the Greengenes database and are not officially accepted by the Society for General Microbiology. HET, *Tcra*^−/+^ mice not exposed to repeated water-avoidance stress (rWAS); CON, *Tcra*^−/−^ mice not exposed to rWAS; RES, *Tcra*^−/−^ mice resistant to rWAS-induced colitis, SEN, *Tcra*^−/−^ mice sensitive to rWAS-induced colitis.

As in the B/c mice, the composition of the colonic microbiota of B6-CON mice (*n* = 5) was different from that of B6-HET mice (*n* = 5). The relative abundance of several genera of bacteria that were predominant in the colonic microbiota of B6-HET mice was significantly decreased in the B6-CON mice (e.g., unclassified genera of family S24-7, genus *Allobaculum*, and genus *Turicibacter*; FDR-corrected *P* value = 0.03) (Fig 2A). At the phylotype level, the relative abundance of many OTUs was significantly different (FDR-corrected *P* < 0.05) in the colonic microbiota of B6-HET mice compared with in that of B6-CON mice (Fig 3). Although species richness was not significantly distinguishable between B6-CON and B6-HET mice (*P* = 0.156) (Fig 2B), the average unweighted UniFrac distance for the colonic microbiota of B6-CON mice was significantly larger (*P* < 0.05) than that for B6-HET mice (Fig 2C). In a principal-coordinates analysis plot, B6-HET mice were clustered together whereas B6-CON mice were more dispersed (Fig 2D). These results indicated that the *TCRα* knockout affected the community structure and stability of the colonic microbiota in the colonic content of B6 mice.

### Exposure to rWAS induced significant changes in the colonic microbiota of B6-*Tcra*^*−/−*^ mice

Exposure to LFW or HFW altered the composition of the colonic microbiota of B6 *Tcra*^−/−^ mice (data not shown) and the composition of the colonic microbiota of B6-LFW and B6-HFW mice was highly variable. To clarify the relationship between the colonic microbiota and exacerbation of colitis, we combined the data for the B6-LFW and B6-HFW mice and then divided the mice into two groups based on histologic score (S1 Table) to produce a group of mice sensitive to rWAS-induced colitis (B6-SEN, *n* = 5) and a group of mice resistant to rWAS-induced colitis (B6-RES, *n* = 5). The composition of the colonic microbiota of B6-SEN mice was significantly different from that of B6-CON mice (Fig 2A); the relative abundance of genus *Clostridium* was significantly increased (>1000-fold; FDR-corrected *P* = 0.02) and the relative abundances of genus *Allobaculum*, unclassified genera of family S24-7, family *Lachnospiraceae*, order *Clostridiales*, and family *Rikenellaceae* were significantly decreased (3.5–448-fold; FDR corrected *P* < 0.05). Furthermore, species richness was significantly reduced in B6-SEN mice compared with that in B6-CON and in B6-RES mice (*P* < 0.05) (Fig 2B). Inter-mouse differences in the microbial composition of the colonic microbiota were significantly greater in B6-SEN mice than in B6-CON mice (*P* < 0.05) (Fig 2C and 2D). The colonic microbiota of B6-CON and B6-RES mice were comparable in terms of bacterial composition and species richness (Fig 2A–2D). Together, these data indicate that the impact of exposure to rWAS varied among B6-*Tcra*^−/−^ mice, that the frequency of exposure to rWAS did not have an effect, and that the microbial community of B6-SEN mice was markedly altered by rWAS.

### Exposure of B6-*Tcra*^−/−^ mice to rWAS induced changes in the relative abundance of several OTUs

Statistically significant changes (FDR-corrected *P* < 0.05) in the relative abundance of several OTUs were observed in the colonic microbiota of B6-SEN mice compared with that of B6-CON mice (Fig 3). The OTUs that were significantly decreased were mainly in the order *Bacteroidales*, especially within the families S24-7 and *Rikenellaceae*, whereas those that were significantly increased were mainly in the order *Clostridiales*.

The detection ratio and relative abundance of the five most abundant OTUs that were significantly increased in the colonic microbiota of B6-SEN mice were determined for each OTU for each group of mice (Table 2). OTUs 4409730, 4481624, and 4425571, which had high similarity to *Clostridium bifermentans*, *Clostridium disporicum*, and *Escherichia coli*, respectively, were detected in both B6 and B/c mice. However, OTUs 4445673 and 1142110, which had high similarity to *C*. *perfringens* and *Clostridium sordellii*, respectively, were detected only in B6 mice, not in B/c mice, and were detected regardless of whether *Tcra* had been knocked out or not. Furthermore, exposure to rWAS increased the relative abundances of these OTUs. Therefore, the expansion of bacterial populations in B6 mice in the normal state, which may have included formerly minor populations, was increased in mice in which colitis was also aggravated.

**Table 2:** Detection ratio and mean abundance of operational taxonomic units (OTUs) significantly increased in *Tcra*^−/−^ mice exposed to repeated water-avoidance stress.

		OTU 4481624		OTU 4409730		OTU 4425571		OTU 4445673		OTU 1142110	
		*Clostridium disporicum*		*Clostridium bifermentans*		*Escherichia coli*		*Clostridium perfringens*		*Clostridium sordellii*	
Background		Detection ratio	Mean abundance	Detection ratio	Mean abundance	Detection ratio	Mean abundance	Detection ratio	Mean abundance	Detection ratio	Mean abundance
**BALB/c**	**HET (*n* = 5)**	100%	0.017%	100%	0.033%	80%	0.004%	0%	n.d.	0%	n.d.
	**CON (*n* = 5)**	100%	0.032%	100%	0.344%	100%	0.145%	0%	n.d.	0%	n.d.
	**LFW (*n* = 4)**	75%	0.025%	100%	2.011%	100%	0.036%	0%	n.d.	0%	n.d.
	**HFW (*n* = 5)**	100%	0.013%	100%	0.799%	100%	0.005%	0%	n.d.	0%	n.d.
**C57BL/6**	**HET (*n* = 5)**	100%	0.053%	100%	0.012%	40%	0.002%	80%	0.010%	100%	0.004%
	**CON (*n* = 5)**	100%	0.006%	60%	0.006%	20%	0.001%	80%	0.004%	20%	0.002%
	**RES (*n* = 5)**	100%	0.340%	100%	2.110%	100%	0.032%	100%	1.172%	100%	0.079%
	**SEN (*n* = 5)**	100%	21.661%	100%	6.122%	100%	0.931%	100%	6.933%	100%	2.847%

n.d., not detected.

HET, *Tcra*^−/+^ mice not exposed to repeated water-avoidance stress (rWAS); CON, *Tcra*^−/−^ mice not exposed to rWAS; LFW, *Tcra*^−/−^ mice exposed to low-frequency (1 day) rWAS; HFW, *Tcra*^−/−^ mice exposed to high-frequency (5 days) rWAS; RES, *Tcra*^−/−^ mice resistant to rWAS-induced colitis; SEN, *Tcra*^−/−^ mice sensitive to rWAS-induced colitis.

### PLC-producing *Clostridium* species increased in B6-*Tcra*^−/−^ mice in which colitis was aggravated

*Clostridium perfringens* and *C*. *sordellii* produce the extracellular enzyme PLC [34]. PLC is a major virulence factor that is toxic to mammals because it hydrolyzes phosphatidylcholine and sphingomyelin, which are important constituents of the eukaryotic cell membrane [35]. The relative abundance of OTU 4445673 (which was assigned to *C*. *perfringens*) and 1142110 (which was assigned to *C*. *sordellii*) were correlated weakly positively with the histological score (OTU 4445673; *r* = 0.392, *P* = 0.26, OTU 1142110; *r* = 0.559, *P* = 0.09, and sum of both OTU; *r* = 0.438, *P* = 0.21). To confirm the presence of PLC-producing *Clostridium* species in the colonic microbiota of B6 mice, we spread diluted colonic contents on selection agar and isolated PLC-positive colonies. The 16S rRNA gene sequence of the isolate was identical to that of OTU 1142110 and had 99.6% similarity with that of *C*. *sordellii*, indicating that OTU 1142110 represents PLC-producing *C*. *sordellii*.

**Fig 4:**
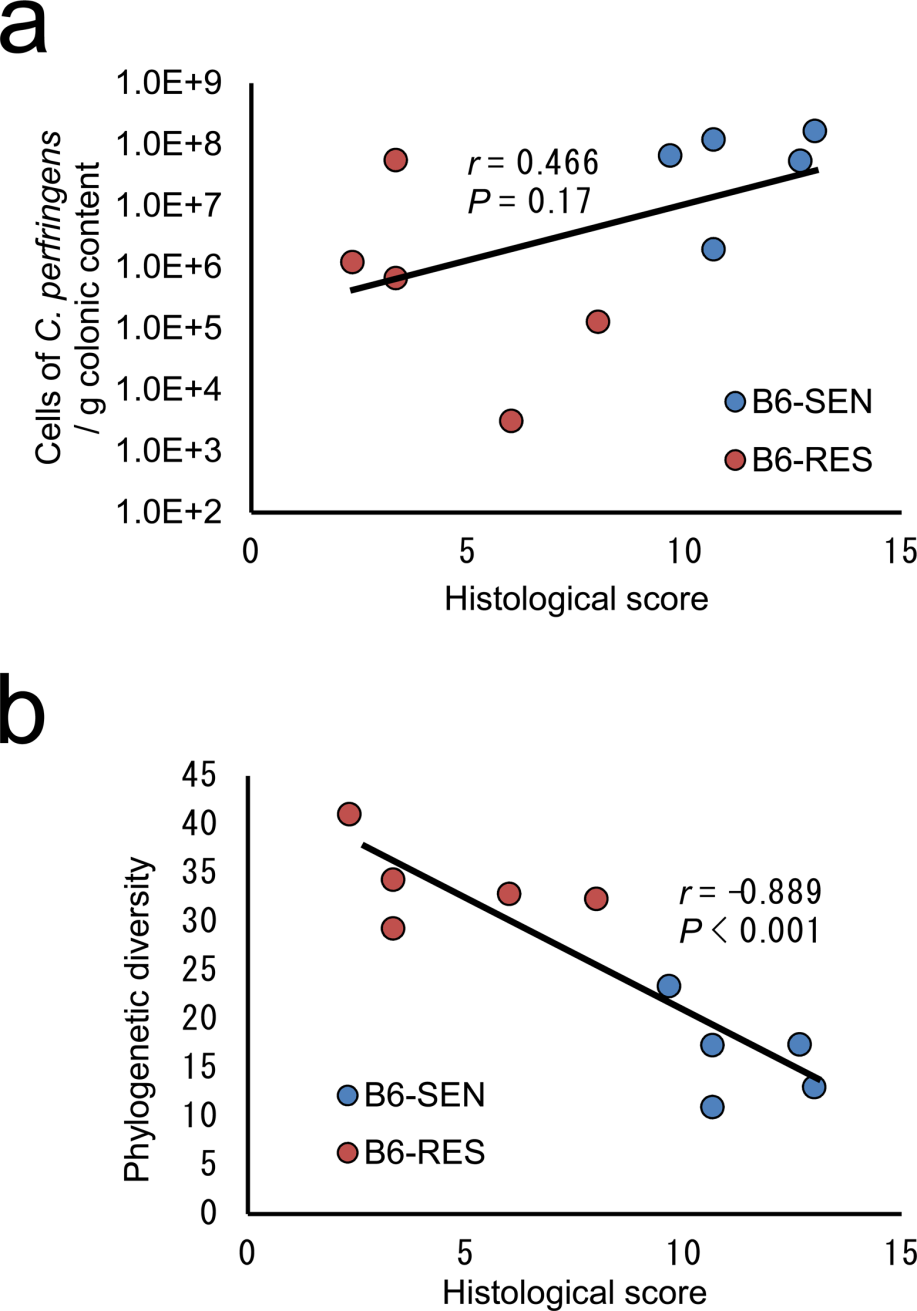
Correlation between histological score and concentration of *Clostridium perfringens* or phylogenetic diversity. (a) *C*. *perfringens* was quantified in colonic contents by means of quantitative PCR. The concentration of *C*. *perfringens* was positively correlated with histological score; however, this was not statistically significant (*P* = 0.17). (b) Phylogenetic diversity was calculated based on 75 000 reads per sample. The phylogenetic diversity of colonic microbiota was significantly negatively correlated with the histological score (*P* < 0.001). RES, *Tcra*^−/−^ mice resistant to rWAS-induced colitis; SEN, *Tcra*^−/−^ mice sensitive to rWAS-induced colitis.

Next, to confirm the presence of PLC-producing *C*. *perfringens* in the colonic microbiota of B6 mice, we performed quantitative PCR targeting the *C*. *perfringens*–specific PLC-encoding gene. *Clostridium perfringens* was detected in the colonic samples from all of the B6-SEN and B6-RES mice, and the concentration of *C*. *perfringens* in the colonic contents was weakly positively correlated with the histological score (*r* = 0.466, *P* = 0.17) (Fig 4A). Of the mice with a large population of *C*. *perfringens*, several had a lower histological score than the others and were found to have a phylogenetically diverse microbiota. Furthermore, phylogenetic diversity was strongly negatively correlated to histological score regardless of the size of the population of *C*. *perfringens* (*r* = −0.889, *P* < 0.001) (Fig 4B).

### Concentrations of free IgA in mouse luminal contents was increased after exposure to rWAS

**Fig 5:**
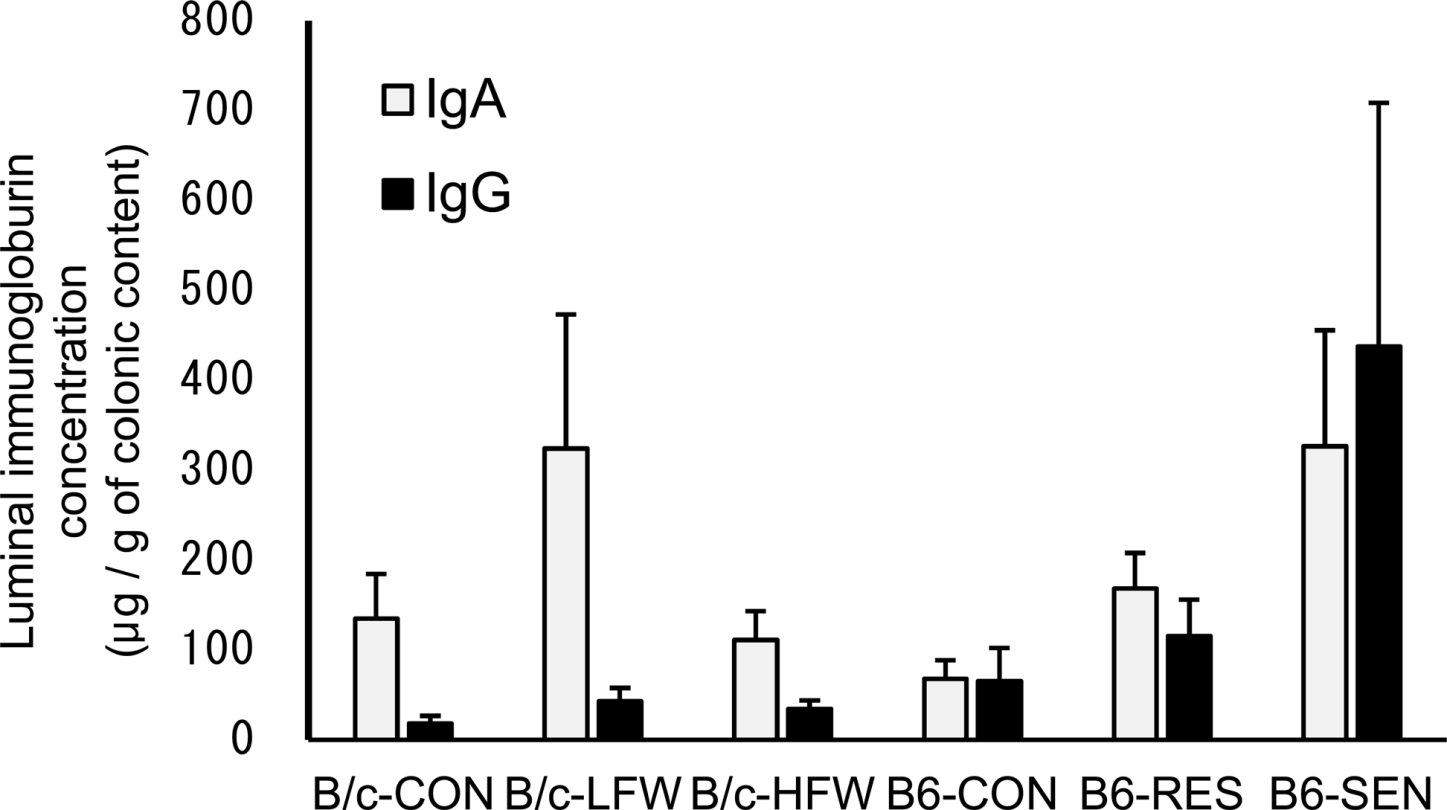
Concentrations of luminal IgA and IgG in *Tcra*^−/−^ mice exposed to repeated water-avoidance stress (rWAS). Concentrations of total IgA and IgG in the colonic content of each mouse were measured by using a sandwich enzyme-linked immunosorbent assay. Data are presented as mean + standard deviation. CON, *Tcra*^−/−^ mice not exposed to rWAS; LFW, *Tcra*^−/−^ mice exposed to low-frequency (1 day) rWAS; HFW, *Tcra*^−/−^ mice exposed to high-frequency (5 days) rWAS; RES, *Tcra*^−/−^ mice resistant to rWAS-induced colitis; SEN, *Tcra*^−/−^ mice sensitive to rWAS-induced colitis.

Intestinal secretory immunoglobulins may be essential for the maintenance of the symbiotic balance between the colonic microbiota and the host immune system. Since the amount and quality of luminal secretory IgA has been shown to influence the diversity and phylogenetic structure of the GI microbiota [36], changes in the secretory immunoglobulin profile were expected to occur and to be correlated with changes in the composition of the colonic microbiota. In B/c mice, the concentrations of luminal free IgA and IgG is not significantly elevated in the B/c-LFW or B/c-HFW mice compared to B/c-CON mice. In B6 mice, the concentrations of luminal IgA increased significantly in the B6-SEN mice compared to B6-CON mice (*P* < 0.05), whereas luminal IgG was not significant (*P* = 0.222) (Fig 5). These results indicated that exposure to rWAS increased the secretion of, or reduced the antigen-binding specificity of IgA, and the severity of colitis associated with the concentration of luminal IgA.

## Discussion

### Impact of *Tcra* knockout on the murine colonic microbiota

*Tcra* knockout mice have several dysfunctions of the mucosal immune system that result in the development of severe colitis-like ulcerative colitis [37]. A recent study has shown that the balance of the GI microbiota is maintained by the host mucosal immune system via molecules such as intestinal secretory IgA, which undergoes hypermutation in its heavy and light chains [38]. Our present results indicate that in both B/c and B6 mice, knockout of *Tcra* disrupts these microbiota balancing systems, resulting in an unstable microbiota (Figs 1 and 2). Since the loss of T cell affects the bacterial composition (36), the difference between HET mice and CON mice might be to a great extent based on the lack of the *Tcra*-related immune response, rather than the cage effect. Compared with *Tcra*^−/+^ mice, the relative abundances of unclassified genera of family S24-7 were reduced, especially in B6-*Tcra*^−/−^ mice (Figs 2A and 3). The members of family S24-7 are ubiquitous, predominant components of the mouse luminal contents [39–42], and a decrease of this population may be correlated with the presence of inflammatory conditions such as diabetes [40], obesity [41], dextran sodium sulfate–induced colitis [42], and the rWAS-induced colitis observed in the present study (Fig 2A). *Allobaculum*, a member of the family *Erysipelotrichaceae*, was detected at a high relative abundance in the colonic microbiota of B6-HET mice, but this population was reduced in B6-CON mice (Fig 2A). According to previous reports, decreases in the relative abundance of family *Erysipelotrichaceae* (including *Allobaculum*) is correlated with inflammation caused by factors such as a high-fat diet [43], obesity [41], and the absence of normal mucus production [44]. In specific pathogen-free mice, members of the families S24-7 and *Erysipelotrichaceae* have been shown to be coated by secretory IgA *in vivo* [45]. Therefore, the disruption of the mucosal immune system caused by *Tcra* knockout may reduce the relative abundances of members of the colonic microbiota that are normally coated by IgA, which might be crucial for balancing the GI microbiota.

### Factors affecting the response to exposure to rWAS in B/c and B6 mice

Although the effect of *Tcra* knockout was comparable in B/c and B6 mice, the response to rWAS was markedly different (S1 Table). B/c mice were more tolerant than B6 mice of rWAS-associated colonic inflammation. A similar trend was observed in a previous study that showed that chronic stress–induced diarrhea was more easily induced in B6 mice than in B/c mice, and that this was associated with a higher concentration of stress-elicited colonic 5-hydroxytryptamine in the B6 mice [17]. Our present data suggest that the expansion of B6-specific populations of pathogens such as *C*. *perfringens* and *C*. *sordellii* may be one factor that defines the degree of aggravation of colitis in B6-*Tcra*^−/−^ or B/c-*Tcra*^−/−^ mice. *Clostridium perfringens* and *C*. *sordellii* are both pathogens that produce the major virulence factor PLC, and the populations of these bacteria were increased in the mice that developed severe colitis. Therefore, the expansion of specific populations of pathogens in the normal state, possibly even including formerly minor populations, may affect the degree of aggravation of colitis.

### Microbial community in the colonic microbiota of B6-SEN mice

The relative abundances of many groups of bacteria in the colonic microbiota of B6-SEN mice were markedly different from those in B6-CON mice. In addition to the putative B6-specific pathogens already discussed, the populations of OTUs 4409730, 4481624, and 4425571, which had high similarity to *C*. *bifermentans*, *C*. *disporicum*, and *Escherichia coli*, respectively, were expanded in B6-SEN mice. Although *C*. *bifermentans* also produces PLC [46], because of the low 16S rRNA gene sequence similarity to *C*. *bifermentans* (95%), the function of OTU 4409730 is unclear. The pathogenicity of *C*. *disporicum* has not been well studied. The population of OTU 4425571, which was assigned to the family *Enterobacteriaceae*, increased in B6-SEN mice. The relative abundance of *Enterobacteriaceae* is known to be increased in patients with gastrointestinal diseases such as colorectal cancer and IBD, and an increased *Enterobacteriaceae* population may be a general indicator of a disrupted intestinal microbiota [47]. Although it remains unclear whether these phylotypes contribute to the exacerbation of colitis or whether they are simply an indicator of dysbiosis, our results suggest that rWAS-induced inflammation may produce an environment that promotes the expansion of *Clostridium* and other facultative anaerobic species. Changes in gut peristalsis or in the redox potential of the colonic contents caused by severe mucosal inflammation may contribute to producing such an environment. On the other hand, in the present study, the populations of many phylotypes, mainly those belonging to the order *Bacteroidales*, especially those in family S24-7, contracted after exposure to rWAS. However, it is hard to determine the physiological function and ecological role of these bacteria in the gut because their 16S rRNA gene sequence had less than 90% similarity with known species. Therefore, further isolation and characterization of these phylotypes is needed to determine whether they are protective or whether they are an indicator of a disrupted intestinal microbiota.

### Relationship between bacterial diversity and colitis mediated by immunoglobulins

Loss of diversity in the GI microbiota has been reported in patients with IBD and in animal models of colitis such as IL10-deficient mice [48], as well as in several strains of immunodeficient mice [36]. Consistent with these previous reports, the phylogenetic diversity of the colonic microbiota of B6-SEN mice decreased significantly with exacerbation of colitis (Fig 4A) and the concentration of pathogenic *C*. *perfringens* in the colonic contents increased in the mice with exacerbated colitis, suggesting that increases in the relative abundance of *C*. *perfringens* may be one aggravating factor in colitis. Interestingly, the mice with high phylogenetic diversity did not develop colitis despite possessing relatively large populations of *C*. *perfringens*. These results indicate that the loss of phylogenetic diversity in the colonic microbiota is a key factor in, or important indicator of, the development of colitis. B6-SEN mice also showed the increasing of luminal IgA compared to B6-CON mice, indicates that chronic stress-induced activation of the hypothalamic–pituitary–adrenal axis might affect the differentiation of B cells or the integrity of the mucosal barrier function. A symbiotic regulatory loop between the gut microbiota and the host immune system has been reported; rich and balanced bacterial communities have been shown to induce Foxp3^+^ follicular T cells and the differentiation of IgA-producing plasma cells, and IgA has been shown to regulate the diversity and composition of the microbiota [36]. Therefore, the T cell dependent immune response helps keeping the bacterial diversity higher, and *Tcra* knockout likely caused dysfunction of this symbiotic regulatory loop, which amplified the effect of stress on the colonic microbiota. However, the detailed mechanism of the regulation of the GI microbiota via IgA remains unclear. In addition, the role of IgG with respect to the microbiota is not well understood. Revealing the detailed molecular mechanism of the contributions of not only IgA but also IgG to the maintenance of phylogenic diversity in the colonic microbiota will provide useful information for preventing stress-related increases in disease activity in IBD.

## Conclusion

Long-term exposure of *Tcra*^−/−^ mice to rWAS altered the composition of the colonic microbiota of B6 mice, which resulted in the development of severe colitis. B6 and B/c mice showed a strain-specific response to rWAS, and the expansion or contraction of populations of pathogens such as *C*. *perfringens* and *C*. *sordellii*, which both produce PLC, might play an important role in this response. Furthermore, the phylogenic diversity of the colonic microbiota was strongly negatively correlated with the degree of aggravation of colitis, and probable stress-induced activation of the hypothalamic–pituitary–adrenal axis affected IgA production. These results suggest that disruption of the system that balances the colonic microbiota and expansion of specific populations of endogenous pathogens are key factors in the development of stress-induced aggravation of colitis. Importantly, although some members of the genus *Clostridium* were commonly increased in mice with severe colitis, the microbial composition of the colonic microbiota showed high inter-mouse variability. This suggests that the bacterial community structure can shift even in a controlled, stable breeding environment, which indicates the difficulty in revealing a single etiological pathogenic species responsible for IBD. In addition, mice with a phylogenetically diverse microbiota did not show exacerbation of colitis despite possessing a relatively high abundance of *C*. *perfringens*. Therefore, our results suggest that preserving a high phylogenetic diversity in the colonic microbiota might be an important factor for preventing increases in disease activity triggered by chronic psychological stress in IBD.

## Supporting Information

S1 Table Sample details. (DOCX)

S2 Table The relative expression level of cytokines and stress-related mRNA in LI-LPMC in *Tcra*^-/-^ mice. Large intestinal lamina propria mononuclear cells (LI-LPMCs) were isolated from mice. Total mRNA was purified from collected LI-LPMCs. Reverse transcriptional polymerase chain reaction was performed for each gene. Expression levels of each group were standardized based on that of the CON group.
(DOCX)
