# Supplementary material for: Chronic Psychological Stress Disrupted the Composition of the Murine Colonic Microbiota and Accelerated a Murine Model of Inflammatory Bowel Disease
Source: PLoS One. 2016 Mar 7;11(3):e0150559. doi: 10.1371/journal.pone.0150559 (PMC4780833; doi:10.1371/journal.pone.0150559)
Supplement: S1 Table — (DOCX) [file pone.0150559.s001.docx]

**S1 Table. Sample details**

| **Background** | **ID** | **TCRα** | **Treatment** | **Histological score** | **Sensitivity to rWAS-induced aggravation of colitis** |
| --- | --- | --- | --- | --- | --- |
| C57BL/6 | B6.1 | -/+ | Control | N.D. | - |
|  | B6.2 | -/+ | Control | N.D. | - |
|  | B6.3 | -/+ | Control | N.D. | - |
|  | B6.4 | -/+ | Control | N.D. | - |
|  | B6.5 | -/+ | Control | N.D. | - |
|  | B6.N1 | -/- | Control | 5 | - |
|  | B6.N2 | -/- | Control | 10.7 | - |
|  | B6.N3 | -/- | Control | 8.3 | - |
|  | B6.N4 | -/- | Control | 3 | - |
|  | B6.N5 | -/- | Control | 2.3 | - |
|  | B6.L1 | -/- | LFW | 3.3 | Resistant |
|  | B6.L2 | -/- | LFW | 9.7 | Sensitive |
|  | B6.L3 | -/- | LFW | 2.3 | Resistant |
|  | B6.L4 | -/- | LFW | 13 | Sensitive |
|  | B6.L5 | -/- | LFW | 10.7 | Sensitive |
|  | B6.H1 | -/- | HFW | 8 | Resistant |
|  | B6.H2 | -/- | HFW | 6 | Resistant |
|  | B6.H3 | -/- | HFW | 3.3 | Resistant |
|  | B6.H4 | -/- | HFW | 12.7 | Sensitive |
|  | B6.H5 | -/- | HFW | 10.7 | Sensitive |
| BALB/c | B/c.1 | -/+ | Control | N.D. | - |
|  | B/c.2 | -/+ | Control | N.D. | - |
|  | B/c.3 | -/+ | Control | N.D. | - |
|  | B/c.4 | -/+ | Control | N.D. | - |
|  | B/c.5 | -/+ | Control | N.D. | - |
|  | B/c.N1 | -/- | Control | 4 | - |
|  | B/c.N2 | -/- | Control | 1 | - |
|  | B/c.N3 | -/- | Control | 4 | - |
|  | B/c.N4 | -/- | Control | 7 | - |
|  | B/c.N5 | -/- | Control | 1 | - |
|  | B/c.L2 | -/- | LFW | 7 | - |
|  | B/c.L3 | -/- | LFW | 7 | - |
|  | B/c.L4 | -/- | LFW | 2 | - |
|  | B/c.L5 | -/- | LFW | 4 | - |
|  | B/c.H1 | -/- | HFW | 6 | - |
|  | B/c.H2 | -/- | HFW | 5 | - |
|  | B/c.H3 | -/- | HFW | 5 | - |
|  | B/c.H4 | -/- | HFW | 6 | - |
|  | B/c.H5 | -/- | HFW | 9 | - |

N.D.: not determined.

TCRα, T cell receptor alpha chain gene; LFW, *Tcra*^−/−^ mice exposed to low-frequency (1 day) rWAS; HFW, *Tcra*^−/−^ mice exposed to high-frequency (5 days) rWAS.
