# Supplementary material for: Chronic Psychological Stress Disrupted the Composition of the Murine Colonic Microbiota and Accelerated a Murine Model of Inflammatory Bowel Disease
Source: PLoS One. 2016 Mar 7;11(3):e0150559. doi: 10.1371/journal.pone.0150559 (PMC4780833; doi:10.1371/journal.pone.0150559)
Supplement: S2 Table — Large intestinal lamina propria mononuclear cells (LI-LPMCs) were isolated from mice. Total mRNA was purified from collected LI-LPMCs. Reverse transcriptional polymerase chain reaction was performed for each gene. Expression levels of each group were standardized based on that of the CON group. (DOCX) [file pone.0150559.s002.docx]

**S2 Table. The relative expression level of cytokines and stress-related mRNA in LI-LPMC in *Tcra*^-/-^ mice.** Large intestinal lamina propria mononuclear cells (LI-LPMCs) were isolated from mice. Total mRNA was purified from collected LI-LPMCs. Reverse transcriptional polymerase chain reaction was performed for each gene. Expression levels of each group were standardized based on that of the CON group.

|  | C57BL/6 | | | BALB/c | | |
| --- | --- | --- | --- | --- | --- | --- |
|  | CON (*n* = 5) | RES (*n* = 5) | SEN (*n* = 5) | CON (*n* = 5) | LFW (*n* = 5) | HFW (*n* = 5) |
| *UCN2* | 1.00±0.44 | 6.35±2.03* | 1.87±0.43 | 1.00±0.25 | 0.65±0.08 | 0.83±0.05 |
| *CRFR2* | 1.00±0.29 | 2.51±0.53* | 0.81±0.38 | 1.00±0.10 | 0.90±0.23 | 1.93±0.51 |
| *IFN-γ* | 1.00±0.25 | 2.06±0.53 | 4.69±2.90 | 1.00±0.62 | 2.51±0.79 | 1.26±0.42 |
| *IL-6* | 1.00±0.48 | 1.60±0.46 | 0.85±0.42 | 1.00±0.27 | 1.21±0.26 | 0.75±0.13 |
| *TNF-α* | 1.00±0.13 | 1.47±0.52 | 1.42±0.16 | 1.00±0.17 | 0.61±0.12 | 0.90±0.29 |

* *P* < 0.05 (vs. CON group).

CON, *Tcra*^−/−^ mice not exposed to rWAS; LFW, *Tcra*^−/−^ mice exposed to low-frequency (1 day) rWAS; HFW, *Tcra*^−/−^ mice exposed to high-frequency (5 days) rWAS; RES, *Tcra*^−/−^ mice resistant to rWAS-induced colitis; SEN, *Tcra*^−/−^ mice sensitive to rWAS-induced colitis; *UCN2*, urocortin 2; *CRFR2*, corticotropin-releasing factor receptor 2; *IFNγ*, interferon gamma; *IL-6*, interleukin-6; *TNFα*, tumor necrosis factor alpha.
